# Application-Specific Measurement Uncertainty Software for Measuring Enrofloxacin Residue in Aquatic Products Using the Quick Quantitative (QQ) Method

**DOI:** 10.3390/biology15020119

**Published:** 2026-01-07

**Authors:** Bo Rong, Haitao Zhang, Wenjing He, Peilong Song, Yuanyuan Xu, Emmanuel Bob Samuel Simbo, Haizhou Jiang, Liping Qiu, Lei Zhu, Longxiang Fang, Suxian Qi, Tingting Yang, Zhongquan Jiang, Shunlong Meng, Chao Song

**Affiliations:** 1 School of Marine Technology and Environment, Dalian Ocean University, Dalian 116023, China; r2403432988@163.com (B.R.);; 2 Wuxi Fisheries College, Nanjing Agricultural University, Wuxi 214081, China; 3 Freshwater Fisheries Research Center, Chinese Academy of Fishery Sciences, Wuxi 214081, China; 4 Laboratory of Quality & Safety Risk Assessment for Aquatic Products on Environmental Factors (Wuxi), Ministry of Agriculture and Rural Affairs, Wuxi 214081, China; 5 Key Laboratory of Freshwater Fisheries and Germplasm Resources Utilization, Ministry of Agriculture and Rural Affairs, Freshwater Fisheries Research Center, Chinese Academy of Fishery Sciences, Wuxi 214081, China; 6 Jiangsu SuWei Institute of Microbiology Co., Ltd., Wuxi 214063, China; 13485044311@139.com (H.Z.);; 7 East China Sea Fisheries Research Institute, Chinese Academy of Fishery Sciences, Shanghai 200090, China; 8 Key Laboratory of Environmental Health Impact Assessment of Emerging Contaminants, Ministry of Ecology and Environment, School of Environmental Science and Engineering, Shanghai Jiao Tong University, Shanghai 200240, China

**Keywords:** measurement uncertainty, Quick Quantitative method, expanded uncertainty, conformity assessment, aquaculture, enrofloxacin, ciprofloxacin, mobile application

## Abstract

Quick Quantitative (QQ) immunoassays have been increasingly applied for the measurement of enrofloxacin (ENR) and ciprofloxacin (CIP) residues in aquaculture due to their speed and convenience. However, their quantitative reliability remains limited because measurement uncertainty (MU) is rarely considered during field testing. To enhance the metrological reliability of QQ-based residue analysis, we developed AquaUncertainty Pal, a mobile application that embeds real-time MU computation into the QQ workflow. The software automatically evaluates uncertainty sources during sampling and pipetting, visualizes the uncertainty budget, and guides users through optimized operations. The framework was validated against ISO/IEC 17025–accredited LC–MS/MS and assessed through a user study involving 20 frontline technicians. With the integrated software, pipetting precision (RSD) at 100 μL improved from 4.1% to 1.79%, the inter-operator variability (CV) decreased by 52%, and conformity assessment accuracy for samples near the maximum residue limit (MRL) increased from 25% to 70%. This suggests that real-time MU visualization effectively guided technicians toward consistent pipetting and interpretation behavior. These results demonstrate that integrating MU into the QQ workflow is both feasible and effective, substantially improving reliability and providing a replicable digital framework for uncertainty-informed residue monitoring in aquaculture.

## Simple Summary

Rapid tests are widely used to screen antibiotic residues in aquaculture products, but the results can vary because small operational differences (e.g., pipetting and interpretation) introduce uncertainty. We developed a mobile application (AquaUncertainty Pal) that calculates and visualizes measurement uncertainty during the Quick Quantitative (QQ) workflow and provides step-by-step guidance to users. Using a before–after study with frontline technicians and cross-validation against ISO/IEC 17025–accredited LC–MS/MS, we show that real-time uncertainty feedback improves pipetting consistency and reduces operator-to-operator variability. The proposed approach offers a practical way to make rapid on-site residue screening more reliable and more comparable across technicians and testing sites.

## 1. Introduction

Aquatic products play a crucial role in global food supply, particularly in countries with intensive aquaculture production such as China. However, the widespread use of veterinary antibiotics to prevent bacterial diseases has raised growing concerns about drug residues and food safety [1,2]. Regulatory agencies, including the European Union and China’s Ministry of Agriculture, have therefore established strict maximum residue limits (MRLs) for fluoroquinolones such as enrofloxacin (ENR) and ciprofloxacin (CIP) in fishery products. Ensuring compliance with these limits requires analytical methods that are not only sensitive and accurate but also feasible for field use [3].

Enrofloxacin (ENR) is a fluoroquinolone antibiotic widely used in aquaculture for the prevention and treatment of bacterial infections, and ciprofloxacin (CIP) is its major metabolite and/or a closely related fluoroquinolone that is frequently considered in residue monitoring. Because these compounds may persist in edible tissues and are regulated by maximum residue limits (MRLs), reliable screening and confirmatory testing are essential for food safety and compliance. The chemical structures of ENR and CIP are provided in Figure S12.

Laboratory-based methods such as liquid chromatography–mass spectrometry (LC–MS/MS) are recognized as the gold standard for quantitative residue analysis, providing excellent sensitivity and selectivity [4]. Nevertheless, these methods require expensive instruments, trained personnel, and long turnaround times, which limit their applicability in on-site measuring. As a complementary approach, immunochromatographic strip assays and Quick Quantitative (QQ) immunoassays have been widely adopted for rapid residue detection [5]. Although QQ assays offer speed and convenience, their quantitative reliability is often questioned, particularly when test results are close to the MRL. The major reason is that measurement uncertainty (MU) an essential metrological concept is rarely considered during rapid testing [6].

According to the ISO Guide to the Expression of Uncertainty in Measurement (GUM), measurement uncertainty can be expressed as a combined standard uncertainty ($u_{c}$) and, when required, as an expanded uncertainty (U = k·$u_{c}$) using an appropriate coverage factor (k). ISO/IEC 17025 also requires laboratories to evaluate and report measurement uncertainty for quantitative results, which becomes especially important when screening values are near regulatory decision limits such as MRLs [7,8,9,10,11].

To address this challenge, we developed a mobile application that embeds real-time MU assessment into the QQ detection workflow. The software, named AquaUncertainty Pal, guides users through standardized QQ procedures, records input parameters, calculates MU based on the GUM framework, and visualizes source-specific contributions [4,12,13]. By integrating MU computation into the testing process itself, the approach transforms an abstract metrological concept into a practical decision-support tool [14,15,16]. This study aimed to evaluate the software’s performance in improving operational precision, uncertainty awareness, and decision reliability among frontline technicians, using ENR and CIP detection as representative cases [17,18,19].

## 2. Materials and Methods

### 2.1. Quick Quantitative (QQ) Method Overview

Fish muscle samples used as matrix material were obtained from local sources (aquaculture ponds and/or retail markets). Samples were transported on ice and stored at −20 °C until analysis.

The analytical approach used is a competitive fluorescence immunochromatographic assay designed to measure enrofloxacin (ENR) and ciprofloxacin (CIP) residues. In this system, the target analytes compete with fluorescently labeled monoclonal antibodies at the test line, producing an inverse relationship between analyte concentration and fluorescence intensity higher analyte levels result in lower signal output. This configuration forms the foundation of the Quick Quantitative (QQ) method [5], offering a stable and consistent platform for evaluating measurement uncertainty.

**Figure 1:**
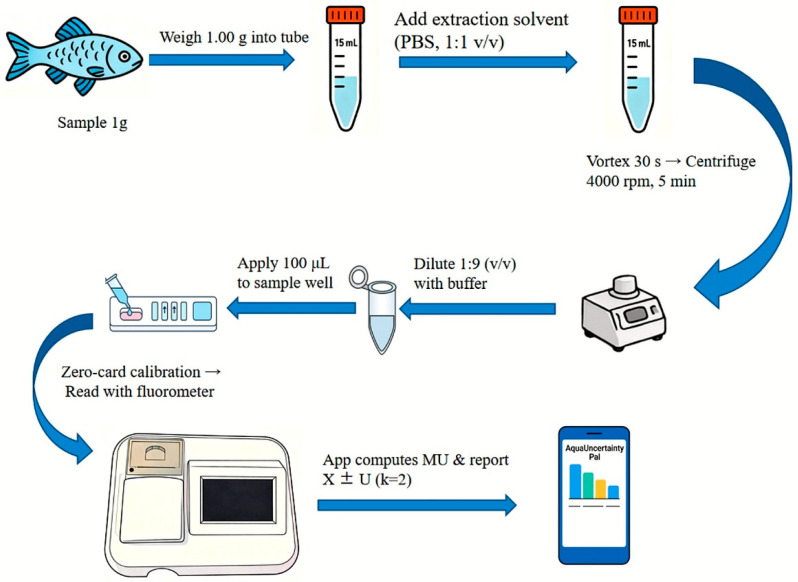
Integrated QQ workflow and embedded measurement uncertainty framework.

The procedures were conducted in line with established standards and tightly integrated with the supporting software. To begin, samples were homogenized to ensure they accurately represented the bulk material. A 1 g portion was precisely weighed and extracted using a methanol–phosphate-buffered saline (PBS) solution, followed by centrifugation at 4000 rpm for 5 min to obtain the supernatant. This extract was diluted at a ratio of 1:9 (*v*/*v*), and 100 μL of the diluted solution was then applied to the test strip’s sample well. The strips were incubated at 24 ± 2 °C for 10 min and measured using a portable fluorescence reader (Jiangsu SuWei Institute of Microbiology Co., Ltd., Wuxi, China). Throughout this process, the integrated software provided real-time prompts, verified essential inputs, and ensured traceability and consistency. The entire analytical workflow from sample preparation and weighing to data validation is summarized in Figure 1. Representative in-app screenshots corresponding to each operational step are provided in the Supporting Information (Figures S1–S11).

This method is capable of accurately measuring antibiotic residues within the 0–300 μg/kg range, meeting the standard requirements for routine monitoring of fluoroquinolones in aquatic food products. All instruments were properly calibrated to maintain analytical precision: the portable fluorometer (Jiangsu SuWei Institute of Microbiology Co., Ltd., Wuxi, China) operated within the 0–300 μg/kg range with a display resolution of 0.01 μg/kg; pipettes with volume ranges from 10 to 100 μL and from 100 to 1000 μL were certified; and sample pre-treatment was supported by a microcentrifuge capable (TDZ5-WS, Xiangyi, Changsha, China) of reaching 10,000 rpm, complemented by a vortex mixer (SN-VORTEX-1E, Shangyi, China). Together, these tools ensure consistent sample processing and mixing, providing dependable data for uncertainty analysis and operator training.

### 2.2. LC–MS/MS Confirmation Method

Confirmatory analysis was performed using an ultra-performance liquid chromatography system coupled to a triple-quadrupole mass spectrometer (UPLC–MS/MS; Xevo TQD, Waters, Milford, MA, USA) operating in electrospray ionization positive mode (ESI+). Chromatographic separation was achieved on a short UPLC column (particle size 1.8 μm, 2.1 × 50 mm). The column temperature was maintained at 35 °C and the sample manager temperature was set at 25.5 °C.

The mobile phases were (A) water containing 0.1% formic acid and (B) methanol containing 0.1% formic acid. The flow rate was 0.300 mL/min and the injection volume was 5 μL. The gradient program (total run time 6.0 min) was as follows: 0.00 min, 80% A/20% B; 0.50 min, 60% A/40% B; 4.50 min, 5% A/95% B; 4.60 min, 5% A/95% B; 4.80 min, 80%A/20% B; 6.00 min, 80% A/20% B.

The LC–MS/MS acquisition function settings and the programmed MRM channels (as displayed in the instrument method interface) are summarized in Supplementary Table S5. Additional details on the LC–MS/MS uncertainty reporting framework and calculation components used in this study are provided in Supplementary Section S5.

Quantification was performed using matrix-matched calibration standards prepared in blank matrix extract. The formulation of calibration-related uncertainty components and the harmonized reporting format (X±U, k=2) used for LC–MS/MS results are described in Supplementary Section S5.

### 2.3. Measurement Uncertainty (MU) Model

To keep the computations trustworthy, the internal uncertainty modules of the software implement the mathematical rules set out in the Guide to the Expression of Uncertainty in Measurement (GUM) [7]. Each module was stringently cross-checked against a reference Excel implementation; differences were under 0.01%, confirming the precision and stability of the algorithms.

The combined standard uncertainty is assembled from five main contributors: Sample Weighing Uncertainty, Extractor Uncertainty, zero-card Calibration Uncertainty, Curve Fitting Uncertainty, and Lab Card Uncertainty. The extractor and zero-card calibration terms are particularly sensitive to pipetting performance, while the Lab Card term is a composite that captures strip-to-strip variability, sample application, and signal reading. Multi-batch testing on the same sample produced a test-strip relative standard deviation (RSD) of 4.5%, which aligns closely with the 4% RSD specified by the manufacturer, supporting the validity of that parameter.

Individual standard-uncertainty components were combined into the overall combined standard uncertainty $\left(u_{c}\right)$ using the law of propagation of uncertainty (the root-sum-of-squares method). The calculation is expressed as:

(1) $$u_{c}(y)=\sqrt{\sum_{i=1}^{N}{\left[c_{i}u(x_{i})\right]}^{2}}.$$

where $u_{c}\left(y\right)$ is the combined standard uncertainty of the final measurement result, y; $u\left(x_{i}\right)$ represents the standard uncertainty of each input quantity, $x_{i}$ and $c_{i}$ is the corresponding sensitivity coefficient, which describes the extent to which the output, y is influenced by changes in the input, $x_{i}$. For the additive model used in this study, all sensitivity coefficients were assigned a value of 1.

In this study, RSD was used as the primary precision metric for specific steps (e.g., pipetting) and to quantify pre-/post-training improvements.

(2) $$RSD=\frac{SD}{\bar{X}}\times 100\%$$

Here, SD represents the standard deviation obtained from repeated measurements of the same sample, while $\bar{X}$ denotes the mean of those results. The Relative Standard Deviation (RSD) reflects how widely the measurement values vary relative to their mean and is a widely used indicator of operational repeatability and consistency among different technicians. In this study’s uncertainty budget, RSD was employed to quantify how pipetting precision and batch-to-batch variability of test strips contribute to the overall uncertainty. Comparing RSD values before and after operator training clearly demonstrated the software’s effectiveness in enhancing technical accuracy and measurement consistency.

Because the measurement model follows an additive form, all sensitivity coefficients were assigned a value of 1. The combined standard uncertainty was obtained by the law of propagation of uncertainty (root-sum-of-squares), and the expanded uncertainty was $U=k\cdot u_{c}$ with k=2. The five sources used throughout this paper are kept exactly as labeled in our figures and text: Sample Weighing Uncertainty, Extractor Uncertainty, zero-card Calibration Uncertainty, Curve Fitting Uncertainty, and Lab Card Uncertainty. Results are reported as X±U (μg/kg, k=2) [8]. See Table S4 for the source-resolved budget.

To provide a result that can be readily interpreted for conformity assessment, the expanded uncertainty (U) was subsequently calculated [8]. The expanded uncertainty defines an interval about the measurement result that is expected to encompass a large fraction of the distribution of values that could reasonably be attributed to the measurand. It is calculated as:

(3) $$U=k\cdot u_{c}$$

**Figure 2:**
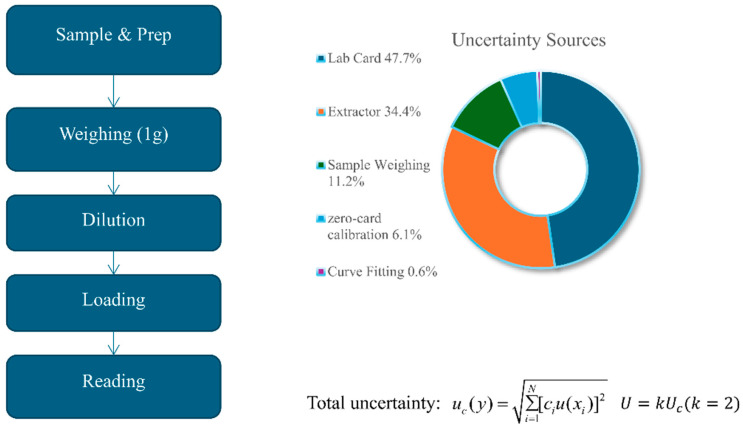
Source-resolved uncertainty budget and step mapping in the QQ workflow.

In accordance with international guidelines (ISO/IEC Guide 98-3) [7], a coverage factor (*k*) of 2 was selected, which for a normal distribution provides a level of confidence of approximately 95%. The final combined standard uncertainty is aggregated from five primary sources, which are qualitatively modeled in our uncertainty framework (Figure 2).

The left panel outlines the seven experimental steps, while the right panel illustrates the relative contributions of the five main uncertainty sources: Lab Card (47.7%), Extractor (34.4%), Sample Weighing (11.2%), zero-card Calibration (6.1%), and Curve Fitting (0.6%). Dashed arrows highlight how each stage in the experimental workflow connects to its associated source of uncertainty. Together, these combined sources measure the expanded uncertainty, which is expressed as X±U, k=2.

### 2.4. Software Architecture Design

The AquaUncertainty Pal software developed in this study adopts a modular architecture consisting of three interconnected components: data input, model calculation, and result output. These modules work together to streamline and standardize the measurement uncertainty (MU) process. The data input module allows technicians to enter essential experimental parameters such as pipette calibration data, sample weight, dilution ratios, and fluorescence readings with built-in validation checks to minimize user errors. The calculation module, built on the GUM framework, performs measurement uncertainty (MU) computations within seconds by integrating operator inputs with pre-defined parameters, ensuring both accuracy and computational efficiency. The result output module presents the final results in the standard “concentration ± U” format and includes visual summaries such as bar charts that display the proportional contribution of each uncertainty source. These visual tools help technicians quickly identify major error contributors and guide targeted process improvements.

The software is developed natively for Android devices using the Kotlin programming language, providing excellent stability and compatibility for on-site use. It operates smoothly on systems with at least Android 8.0, 2 GB of RAM, and 200 MB of storage, which covers most standard portable devices. Numerical computations leverage Kotlin’s built-in libraries for real-time determination of means, RSD, and expanded uncertainty, with future potential for performance upgrades through JNI-based C++ extensions. The interface design follows Google’s Material Design guidelines, featuring structured layouts, uniform typography, and responsive elements for a clean, consistent user experience. Aligned with the assay workflow, the interface provides step-by-step guidance from pipette calibration and sample preparation to data entry and result validation with immediate feedback if errors occur. This integration translates the once abstract concept of MU into a clear, intuitive, and hands-on experience, reducing the learning curve while enhancing the traceability and standardization of on-site testing.

For clarity, the key interfaces of the app-guided workflow are shown in the Supporting Information: activation and login (Figure S1), environmental equilibrium confirmation (Figure S2), sample temperature input (Figure S3), sample weighing and entry (Figure S4), reagent addition volume entry (Figure S5), sample vibration/mixing guidance (Figure S6), calibration curve interface (Figure S7), extraction-solution mixing interface (Figure S8), dilution and strip loading instructions (Figure S9), strip reaction/reading guidance (Figure S10), and the final concentration result interface (Figure S11).

### 2.5. Study Design and Competency Assessment

A before–after paired design included 20 frontline technicians with 3 ± 2 years of experience and no prior MU training. Each participant completed a baseline QQ run (app guidance disabled), a standardized training, and a post-training reassessment. For pipetting precision, each operator performed ten replicates at 100 μL and ten replicates at 1000 μL in one session; RSD was computed per operator. For the conformity assessment task, the regulatory limit (MRL) for total fluoroquinolones (sum of enrofloxacin and ciprofloxacin) in fish muscle was set at 100 µg/kg, in accordance with the China National Food Safety Standard GB 31650-2019 (Maximum Residue Limits for Veterinary Drugs in Food) [20]. Conformity decisions were made based on the “interval-against-MRL” rule: if the entire measurement interval (X±U) was below the MRL, the sample was deemed compliant; if the entire interval was above the MRL, it was deemed non-compliant; and if the MRL fell within the measurement interval, the result was considered inconclusive.The Operator Skill rubric characterized intrinsic skill burden (1 = layperson; 2 = trained on-site technician; 5 = expert analyst).

Technicians completed a standardized training session that combined conceptual instruction (measurement uncertainty, uncertainty sources, and decision rules) with hands-on guided practice using AquaUncertainty Pal. Detailed training materials, questionnaires, and the competency-assessment SOP are provided in Supplementary Section S2.

Training effectiveness was evaluated using a paired pre–post design, including (i) pipetting repeatability tests at 100 μL and 1000 μL and (ii) a structured questionnaire assessing interpretation of “X ± U”, understanding of measurement principles and uncertainty sources, and near-MRL conformity assessment. For near-MRL decisions, correctness was judged against LC–MS/MS ground truth under the interval-against-MRL rule described above.

In the comparative analysis of the QQ method and the LC–MS/MS reference method, a qualitative metric termed “Operator Skill” was used to evaluate the level of expertise required for each method. This metric is scored on a 5-point scale, where a score of 1 signifies a layperson with no training, a score of 2 represents an on-site technician after undergoing the brief, standardized training protocol described in this study, and a score of 5 corresponds to a highly specialized expert, such as a Ph.D.-level analyst with extensive experience in chromatography and mass spectrometry. This parameter assesses the intrinsic complexity and skill threshold of the method itself, rather than the performance of an individual operator.

All assessments and training were conducted under standard laboratory conditions, using reagents and instruments consistent with routine testing to ensure the results are comparable and practically meaningful. Specific measurement uncertainty (MU) indicators and questions are detailed in Supplementary Tables S2 and S3. This segment of the study not only provides a data source for statistical comparison in the Results Section but also offers an objective basis for evaluating the educational and training functions of the software.

### 2.6. Statistical Analysis

According to statistical conventions, we used RSD to quantify within-operator repeatability for specific steps (e.g., pipetting) and inter-operator CV to summarize between-operator variability at the cohort level. Expanded uncertainty was reported as $U=ku_{c}$ (k = 2) to express the metrological interval used in conformity assessment against the MRL, consistent with GUM practice. Before–after comparisons were paired at the operator level. For pipetting RSD (%) at 100 μL and 1000 μL, paired differences were tested after checking normality (Shapiro–Wilk); paired *t*-tests were used if normal and Wilcoxon signed-rank tests otherwise. Changes in inter-operator CV were summarized descriptively and with bootstrap confidence intervals. For near-MRL conformity (correct/incorrect) under the paired design, changes in accuracy were summarized as absolute differences with confidence intervals and tested using McNemar’s test.

## 3. Results

### 3.1. Software Interface and Functional Demonstration

**Figure 3:**
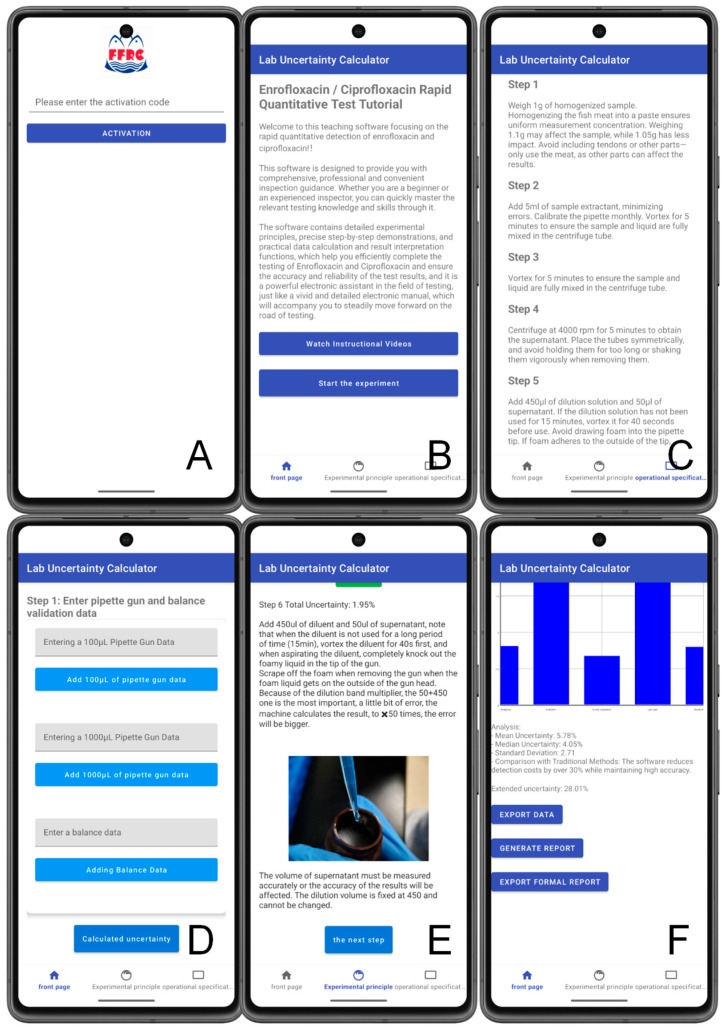
Interface and workflow of AquaUncertainty Pal. (**A**) Home screen and project selection. (**B**) Guided data entry for the QQ assay (sample mass, extraction/dilution factors, and test-card readout). (**C**) Automatic calculation of the measured concentration and expanded uncertainty U (k = 2). (**D**) Source-resolved uncertainty contribution plot showing the relative contribution (%) of key components (sample weighing, extraction/dilution, curve fitting, lab card, and 0-card calibration). (**E**) Built-in validation/export module generating a stepwise calculation record (CSV/Excel) for traceability and cross-checking. (**F**) Technician-training mode that provides standardized prompts and checks to reduce pipetting variability.

The interface design of the AquaUncertainty Pal software aligns with the logic of the QQ method measurement process, incorporating a comprehensive module that spans data entry, operation prompts, and uncertainty visualization (Figure 3). In the data entry interface, technicians must input sample mass, pipette volume, and fluorescence readings. The system automatically verifies whether these input values fall within a reasonable range to prevent systematic errors due to improper operation. The operation prompt module graphically presents the measurement steps, ensuring that experimenters strictly adhere to the standard procedure. Concurrently, the software displays the contribution proportions of various uncertainty sources in real time using bar charts, enabling technicians to intuitively identify the primary error sources. The overall design not only enhances the standardization of the measurement process but also translates the complex concept of MU into intuitive and comprehensible visual information.

### 3.2. Uncertainty Calculation and Methodology Comparison

In the measurement of actual samples, the software automatically completes the uncertainty budget and outputs the results in a standardized format. For instance, the total fluoroquinolone concentration measured in a fish sample was 188.10 ± 23.5 μg/kg (k = 2), corresponding to a 95% coverage interval of 164.6–211.6 μg/kg [21]. The decomposition shows that test-strip batch differences (Lab Card Uncertainty) and Extractor Uncertainty dominate (>70% combined), whereas Sample Weighing, zero-card Calibration, and Curve Fitting Uncertainty contribute less. This result suggests that operator-related steps should be prioritized for control. After targeted training and equipment calibration, the relative contribution of these sources can be further reduced.

**Figure 4:**
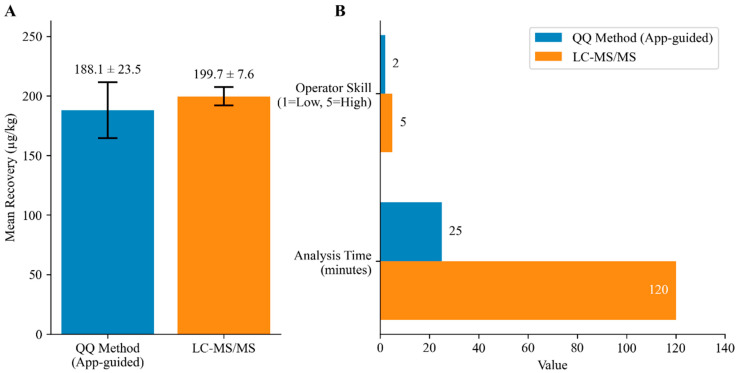
Comparison of the QQ immunoassay and LC–MS/MS for enrofloxacin (ENR) residue determination. (**A**) Mean recovery (µg/kg) obtained by the QQ method (app-guided) and LC–MS/MS from replicate analyses; error bars indicate the expanded uncertainty (U, k = 2) (see Table S1 and Supporting Information Section S5). (**B**) Comparison of required operator skill (1 = low, 5 = high) and analysis time (min) between the QQ workflow and LC–MS/MS.

Although the QQ method showed a higher expanded uncertainty (12.5%), it required only 25 min per sample versus ∼120 min for LC-MS/MS and imposed a substantially lower skill burden. This trade-off is acceptable given the dramatic gain in time efficiency and accessibility for on-site measuring. The quantitative comparison between the QQ immunoassay and LC–MS/MS is shown in Figure 4.

### 3.3. Improvement of Pipetting Precision for Technicians

After standardized training, pipetting precision (RSD) and operational consistency improved across all technicians. Paired differences were approximately normal (Shapiro–Wilk: 100 μL, W = 0.959, *p* = 0.532; 1000 μL, W = 0.974, *p* = 0.843); therefore, two-tailed paired *t*-tests were used. Mean RSD decreased from 4.10% ± 0.68% to 1.79% ± 0.41% at 100 μL (t(19) = 19.26, *p* < 0.001; mean reduction 2.32 percentage points, 95% CI 2.07–2.57; Cohen’s d_z = 4.31) and from 2.52% ± 0.49% to 1.08% ± 0.33% at 1000 μL (t(19) = 20.94, *p* < 0.001; mean reduction 1.45 percentage points, 95% CI 1.30–1.59; Cohen’s d_z = 4.68).

**Figure 5:**
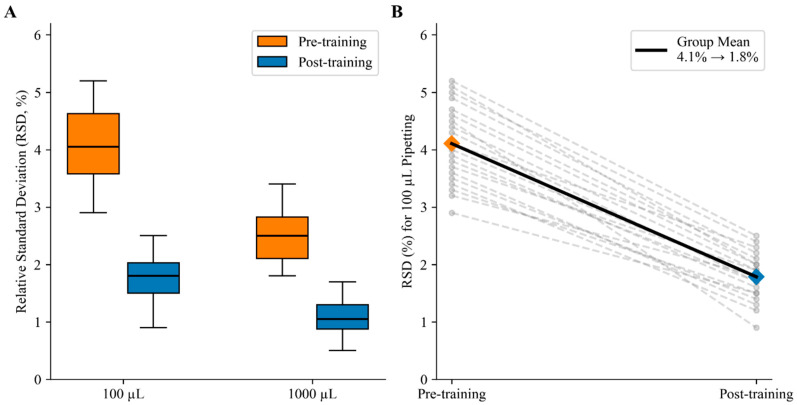
Technician pipetting precision before and after standardized training. (**A**) RSD (%) for 100 µL and 1000 µL pipetting before and after training, summarized across 20 technicians. Boxes indicate the interquartile range with median lines; whiskers denote the 1.5× IQR range. (**B**) Paired change in RSD (%) for 100 µL pipetting from pre-training to post-training. The dotted gray lines represent individual technicians, and the solid black line represents the group mean (4.1% → 1.8%). Normality of paired differences was checked by the Shapiro–Wilk test (*p* > 0.05), and pre- vs. post-training differences were assessed using paired *t*-tests (*p* < 0.001; see Table S2).

Further individual trend analysis showed that all 20 technicians showed a decrease in RSD after training, with no exceptions. The population mean significantly decreased from 4.1% to 1.79% in the 100 μL experiment, and all individuals followed a similar trend (Figure 5). This suggests that software training not only improved the overall level but also narrowed the inter-operator Coefficient of Variation (CV), resulting in a substantial increase in the repeatability and reproducibility of the experiments. The central point is that software training generally improved the precision of pipetting operations and significantly reduced inter-operator and overall experimental variability.

### 3.4. Conceptual Understanding and Conformity Assessment Accuracy

In addition to improving operational performance, the software markedly enhanced the understanding of technicians regarding measurement uncertainty and their ability to make scientifically grounded decisions. As shown in Table 1, the proportion of participants correctly interpreting X ± U results increased from 30% to 80%, indicating substantial improvement in their comprehension of the statistical meaning of measurement results. Similarly, the correct response rate for identifying uncertainty sources and calculation principles rose from 40% to 80%, reflecting deeper conceptual understanding after training. Most notably, for near-MRL samples, the accuracy of conformity assessments increased from 25% to 70%, confirming a significant enhancement in decision reliability. Most notably, for near-MRL samples, the accuracy of conformity assessments against the 100 µg/kg regulatory limit increased from 25% to 70%, conformity decisions followed the interval-against-MRL rule defined in the Methods, and accuracy was evaluated against LC–MS/MS reference results.

## 4. Discussion

The primary contribution of this study is the integration of measurement uncertainty (MU) directly into the QQ method process, facilitated by the mobile software AquaUncertainty Pal for on-site application [9,22]. The results indicate that this framework effectively enhances the reliability of measurement outcomes and offers stronger evidence-based support for frontline technicians in making conformity decisions [10,16,23,24]. Unlike previous QQ method that prioritize speed over uncertainty, this study illustrates that addressing uncertainty is both feasible and essential without compromising efficiency [15,25].

The experimental results clearly indicate that user uncertainty in operational aspects is the primary issue [26]. This is corroborated by our uncertainty decomposition, which reveals that the extraction step accounts for 34.40% of the uncertainty, while weighing and zero-card calibration contribute 11.20% and 6.09%, respectively. These areas are the most error-prone for technicians [17]. The software’s interactive prompts and immediate feedback significantly enhanced technicians’ pipetting precision, reducing the RSD of 100 µL pipetting from 4.1% to 1.79%. Inter-operator variability also narrowed markedly, with the CV decreasing from 18.7% to 9.0% across technicians (see Supplementary Table S2). The improvement observed in all 20 technicians underscores the effectiveness of targeted training and real-time feedback in minimizing human error. Thus, our intervention effectively addressed and reduced the most significant source of error [27,28].

This improvement is also directly reflected in the determination of results. Following the training, the accuracy of judgments made by the technicians regarding the conformity of samples near-MRL increased from 25% to 70% [29,30], and their understanding of “X ± U” improved from 30% to 80% [31]. These results indicate that visualization facilitated consistent interpretation and decision-making near the MRL, as well as a crucial basis for practical judgment by frontline personnel. Such an enhancement holds significant practical importance for food safety supervision, as the frequent issue of “not knowing how to interpret the critical value” often leads to erroneous judgments.

Although this study utilized fluoroquinolones as a model, the results are generalizable [32]. The software framework is modular, dividing total uncertainty into components such as weighing, pipetting, and test strip batch differences, which can be adapted to various measurement systems by adjusting the parameters. For instance, in mycotoxin, pesticide residue, or other veterinary drug testing, the software remains applicable by modifying the RSD and dilution factor of the reagent [33]. This demonstrates that our method is not confined to ENR but offers a generalized framework.

Compared to traditional LC–MS/MS methods, our framework exhibits greater expanded uncertainty; however, it reduces the measurement time for a single sample to just 25 min, as opposed to over two hours for LC–MS/MS. When considering the demands of on-site testing, this efficiency advantage is substantial. The integration of software ensures that increased efficiency does not compromise reliability by omitting measurement uncertainty (MU). Thus, the QQ method effectively balances speed and reliability, meeting the current dual requirements of “fast and reliable” for food safety regulation.

The graphical presentation in the software was crucial in the training process [34]. Many technicians noted in their feedback that the color-partitioned bar graphs of uncertainty provided a visual understanding of the most error-prone parts of the process. This type of visual presentation motivates technicians to enhance their operations more effectively than textual explanations alone. Thus, the software serves as a testing tool as well as a training tool.

This study has several limitations. For instance, the number of samples and application scenarios is limited, primarily focusing on laboratory conditions [35]. Additionally, the current software is designed for a single assay and adapting it to other drugs and assay systems requires further validation. Moreover, the uncertainty model depends on previously accumulated experimental data, and its robustness across different batches and laboratory conditions needs to be tested on a larger scale [36].

Beyond its immediate application in food safety, the success of AquaUncertainty Pal offers valuable insights into the broader fields of Human–Computer Interaction (HCI) and Technology-Enhanced Learning (TEL). From an HCI perspective, the application’s effectiveness arises not only from providing information but also from transforming an abstract, complex concept-measurement uncertainty-into a tangible, visual, and actionable interface [37]. The real-time bar charts that depict the uncertainty budget serve as a powerful cognitive tool, reducing the user’s cognitive load and enabling them to intuitively understand which operational steps have the most impact [38]. In the context of TEL, the application acts as a “digital tutor,” offering situated learning and immediate, corrective feedback [39]. This framework aligns with established pedagogical principles and emphasizes the superiority of active, guided practice over passive instruction for procedural skill acquisition. Consequently, AquaUncertainty Pal can be regarded not merely as a measurement device but as a successful case study in designing cognitive tools that bridge the gap between expert knowledge and novice practice-a framework applicable to other domains requiring quality control in citizen science or decentralized technical work.

In the future, the software’s functionality can be expanded. For instance, the uncertainty of reagents could be dynamically adjusted by incorporating machine learning models that account for environmental factors such as temperature and humidity [40]. Additionally, the computational core could be transferred to PC and server framework to overcome the current batch processing limitations of mobile devices [41]. Furthermore, results could be directly uploaded to the Laboratory Information System (LIMS) via an API interface, enabling seamless integration with regulatory framework. Thus, AquaUncertainty Pal could evolve from a testing software into a distributed monitoring node, becoming part of the future food safety monitoring network [19].

## 5. Conclusions

In this study, measurement uncertainty (MU) was integrated into the routine process of rapid quantitative (QQ) testing and implemented through the mobile software AquaUncertainty Pal. The experimental results indicated that operator uncertainty in key aspects such as extraction, weighing, and zero-card calibration is the primary source affecting the reliability of the assay. The interactive prompts and instant feedback provided by the software effectively mitigate these errors. A user study demonstrated that the pipetting precision (RSD) for 100 μL was significantly improved, inter-operator CV was notably reduced, and conformity assessment accuracy for near-MRL samples substantially improved. The visual display of uncertainty aids technicians in refining their operations, as well as deepens their understanding of concepts such as “X ± U,” thereby serving as a training tool. Compared to the traditional LC–MS/MS method, the QQ method employed in this study offers clear advantages in measurement efficiency. Combined with the uncertainty control offered by the software, it meets the needs of QQ measurement while ensuring reliability. The modular design of the software makes it highly applicable and scalable, allowing it to be extended to other measurement targets, such as pesticide residues, mycotoxins, and other veterinary drugs, by simply adjusting the relevant parameters. Although this study is limited by sample size and application scenarios, and the software’s applicability in different testing systems requires further validation, the overall results demonstrate that embedding uncertainty into the QQ method process is not only feasible but also crucial for enhancing the reliability of on-site testing and the accuracy of regulatory decisions. The proposed framework integrates measurement uncertainty into the QQ immunoassay workflow without compromising efficiency. By quantifying and visualizing uncertainty in real time, AquaUncertainty Pal enhances both analytical reliability and decision confidence in aquatic-product residue monitoring.

## Figures and Tables

**Table 1:** Changes in conceptual understanding and conformity assessment accuracy of technicians before and after training.

Evaluation Metrics	Pre-Training (Baseline)	Post-Training (Outcome)	Improvement
Pipette calibration adherence (%)	45%	88%	43%
Correct Explanation of Measurement Principle (%)	40%	80%	40%
Correct Interpretation of “X ± U” Meaning (%)	30%	80%	50%
Correct Conformity Assessment (%)	25%	70%	45%

## Data Availability

The original data are provided in the article and Supplementary Materials. The AquaUncertainty Pal (Android) installation package used in this study is available for peer review at https://pan.quark.cn/s/fa0d10c0e6fe (accessed on 19 October 2025); for long-term access, readers may contact the corresponding authors.

## Supplementary Materials

The following supporting information can be downloaded at: https://www.mdpi.com/article/10.3390/biology15020119/s1. Figure S1: Activation and login interface; Figure S2: Environmental equilibrium interface; Figure S3: Temperature input interface; Figure S4: Weighing interface; Figure S5: Reagent addition volume entry interface; Figure S6: Vibration interface; Figure S7: Calibration curve interface; Figure S8: Mixing interface; Figure S9: Dilution and spotting instructions; Figure S10: Card reaction interface; Figure S11: Result interface; Figure S12. Chemical structures of enrofloxacin (ENR) and ciprofloxacin (CIP). Table S1. Performance comparison between the Quick Quantitative (QQ) method and the standard LC-MS/MS method; Table S2. Comparison of pipetting precision (RSD %) for 20 technicians before and after training; Table S3. Raw data from multiple independent comparative experiments between LC-MS/MS and Quick Quantitative (QQ) method; Table S4. Source-resolved uncertainty budget for QQ immunoassay; Table S5. Programmed MRM channels and MS parameters for the quinolone LC–MS/MS acquisition function (ESI+), transcribed from the instrument method screen (method label: PAX).
