# Supplementary material for: Application-Specific Measurement Uncertainty Software for Measuring Enrofloxacin Residue in Aquatic Products Using the Quick Quantitative (QQ) Method
Source: Biology (Basel). 2026 Jan 7;15(2):119. doi: 10.3390/biology15020119 (PMC12837475; doi:10.3390/biology15020119)
Supplement: Supplementary file 1 [file biology-15-00119-s001.zip › biology-4038141-supplementary.pdf]

Application-Specific Measurement Uncertainty Software for Measuring Enrofloxacin Residue in Aquatic Products Using the Quick Quantitative (QQ) Method

Bo Rong [1,2,3,4,5,†], Haitao Zhang [6,†], Wenjing He [2], Peilong Song [1,3,4], Yuanyuan Xu [1,3], Emmanuel Bob Samuel Simbo [2,3], Haizhou Jiang [2,3], Liping Qiu [3,4,5], Lei Zhu [2,3,4,5], Longxiang Fang [2,3,4,5], Suxian Qi [6], Tingting Yang [6], Zhongquan Jiang [7,8], Shunlong Meng [1,2,3,4,5,*] and Chao Song [1,2,3,4,5,*]

1 School of Marine Technology and Environment, Dalian Ocean University, Dalian 116023, China; r2403432988@163.com (B.R.); 17639525067@163.com (P.S.); 13369595332@163.com (Y.X.)

2 Wuxi Fisheries College, Nanjing Agricultural University, Wuxi 214081, China;
wj329581123@163.com (W.H.); esimbo58jr@gmail.com (E.B.S.S.); jianghaizhou@ffrc.cn (H.J.); sanshizzz@163.com (L.Z.); fanglongxiang@ffrc.cn (L.F.)

3 Freshwater Fisheries Research Center, Chinese Academy of Fishery Sciences, Wuxi 214081, China; qiulp@ffrc.cn

4 Laboratory of Quality & Safety Risk Assessment for Aquatic Products on Environmental Factors (Wuxi), Ministry of Agriculture and Rural Affairs, Wuxi 214081, China

5 Key Laboratory of Freshwater Fisheries and Germplasm Resources Utilization, Ministry of Agriculture and Rural Affairs, Freshwater Fisheries Research Center, Chinese Academy of Fishery Sciences, Wuxi 214081, China

6 Jiangsu SuWei Institute of Microbiology Co., Ltd., Wuxi 214063, China; 13485044311@139.com (H.Z.); katsu_qi@163.com (S.Q.); tinglin8969@163.com (T.Y.)

7 East China Sea Fisheries Research Institute, Chinese Academy of Fishery Sciences, Shanghai 200090, China; zhongquanj@sjtu.edu.cn

8 Key Laboratory of Environmental Health Impact Assessment of Emerging Contaminants, Ministry of Ecology and Environment, School of Environmental Science and Engineering, Shanghai Jiao Tong University, Shanghai 200240, China

* Correspondence: mengsl@ffrc.cn (S.M.); songc@ffrc.cn (C.S.)

† These authors contributed equally to this work.

This document includes

List of Texts

SECTION S1 Measurement Uncertainty Calculation Principles and App Implementation Details 3

SECTION S2 Instructor Guide for the Laboratory Exercise 5

SECTION S3 Student Experimental Guidance and Data Recording.. 10

SECTION S4 Assessment Materials and Trainee Survey.. 11

SECTION S5 LC–MS/MS Uncertainty Framework and MRM Parameters 15

List of Figure

Figure S1. Activation Function 17

Figure S2. Environmental Equilibrium Page 18

Figure S3. Conditional Temperature Control Page 19

Figure S4. Weighing Interface 20

Figure S5. Extraction Agent Addition 21

Figure S6. Sample Vibration 22

Figure S7. Calibration Curve 23

Figure S8. Mixing the Test Solution 24

Figure S9. Dilution and Spotting Instructions 25

Figure S10. Reagent Card Reaction 26

Figure S11. Concentration Result Page 27

Figure S12. Chemical structures of enrofloxacin (ENR) and ciprofloxacin (CIP).

.... 28

List of Tables

Table S1. Performance comparison between the Quick Quantitative (QQ) method and the standard LC-MS/MS method. 29

Table S2. Comparison of pipetting precision (RSD %) for 20 technicians before and after training.

....30
Table S3. Raw data from multiple independent comparative experiments between LC-MS/MS and Quick Quantitative (QQ) method...31
Table S4. Source-resolved uncertainty budget for QQ immunoassay 32
Table S5. Programmed MRM channels and MS parameters for the quinolone LC–MS/MS acquisition function (ESI+), transcribed from the instrument method screen (method label: PAX). 33

SECTION S1 Pipette and Balance Measurement Uncertainty Calculation and Analysis

Uncertainty Calculation Methods

Pipette Uncertainty Calculation

In this study, the uncertainty calculation of the pipette is based on the standard deviation method of measurement data. The specific calculation steps are as follows:

Data collection: Perform n repeated measurements on a pipette of a certain range to obtain a sequence of measurement values $x_1, x_2, \ldots, x_n$

Mean Value Calculation(S1)：

$$\bar{x} = \frac{1}{n}\sum_{i=1}^{n} x_i \quad \text{(S1)}$$

Calculation of the Sum of Squared Deviations(S2)：

$$\sum_{i=1}^{n}(x_i - \bar{x})^2 \quad \text{(S2)}$$

Calculation of the Standard Deviation(S3)：

$$s = \sqrt{\frac{1}{n-1}\sum_{i=1}^{n}(x_i - \bar{x})^2} \quad \text{(S3)}$$

Calculation of Relative Uncertainty(S4)：

$$U_{\text{pipette}} = \left(\frac{s}{\overline{x}} \right) \times 100\% \tag{S4}$$

Balance Uncertainty Calculation

The method for calculating the uncertainty of the balance is similar to that of the pipette, also based on the standard deviation of the measurement data. The specific steps are as follows:

Data collection: Perform m repeated measurements on the balance to obtain a sequence of measurement values $y_1, y_2, \ldots, y_m$ 。

Mean Value Calculation(S5)：

$$\overline{y} = \frac{1}{m} \sum_{i=1}^{m} y_i \tag{S5}$$

Calculation of the Sum of Squared Deviations(S6)：

$$\sum_{i=1}^{m} (y_i - \overline{y})^2 \tag{S6}$$

Calculation of the Standard Deviation(S7)：

$$s = \sqrt{\frac{1}{m-1} \sum_{i=1}^{m} (y_i - \overline{y})^2} \tag{S7}$$

Calculation of Relative Uncertainty(S8)：

$$U_{\text{balance}} = \left(\frac{s}{\overline{y}} \right) \times 100\% \tag{S8}$$

Calculation of Combined Uncertainty

The combined uncertainty is calculated by combining the uncertainties of each step using the root sum of squares method(S9):

$$U_{total} = \sqrt{U_1^2 + U_2^2 + \ldots + U_n^2} \tag{S9}$$

SECTION S2 Instructor Guide for the Laboratory Exercise

Experimental Objectives

To train trainees to use pipettes and balances for precise measurements and to assess the reliability of measurement results through uncertainty calculations.

Experimental Procedures

1. Pipette Measurement:
- Select a pipette with an appropriate range and perform multiple repeated measurements.
- Record the volume values for each measurement.

2. Balance Measurement:
- While the primary focus of calibration training is on pipettes, trainees will also use the analytical balance for sample weighing and are encouraged to practice repeated weighings (e.g., of a standard weight or a consistent object like a weighing dish) as part of their proficiency development. Record these repeated mass values in Table S1. This practice helps trainees understand the variability inherent in weighing, similar to pipetting. The standard deviation derived from these repeated measurements (or pre-defined balance specifications) is used by the "AquaUncertainty Pal" app to estimate the balance's contribution to the overall measurement uncertainty.

3. Data Processing:
- Process the measurement data using the uncertainty calculation methods mentioned above.
- Calculate and record the relative uncertainties of the pipette and balance.
- Trainees increased their proficiency by training the pipette gun and balance weighing several times, recording the data each time in Table S1.

Training Materials and Preparation

1. Test Kits:

Specified Test Kit: Enrofloxacin/Ciprofloxacin Combined Fluorescent Rapid Test Kit

Jiangsu SuWei Institute of Microbiology Co., Ltd.

Product Batch Number/Item Number: K190242B

Ensure there are sufficient test strips and test fish meat samples. Pay attention to the expiration date.

2. Safety Information and CAS Numbers

• Simulated Samples:

"Practice samples" prepared in advance by the trainer, with known amounts of enrofloxacin and ciprofloxacin added to the fish meat.

- Enrofloxacin CAS: 93106-60-6.
- Ciprofloxacin hydrochloride CAS: 93107-08-5.

3. Instruments and equipment:

Micropipettes (e.g., 10–100 μL, 100–1000 μL) and matching tips.

Smartphone or tablet with the "AquaUncertainty Pal" app installed.

Balance (for the pipette calibration section; high precision is not required, 0.01g is sufficient; the focus is on experiencing the difference).

Common consumables: small tubes, tube racks, markers, timer, trash bin, weighing paper/small dishes.

• Software:

"AquaUncertainty Pal" app.

Acquisition method: pre-install for participants.

Detection Principle

The Enrofloxacin/Ciprofloxacin immunochromatographic fluorescence quantitative detection dual card applies the principle of competitive inhibition immunochromatography. If the sample contains enrofloxacin and ciprofloxacin, they will react with the specific monoclonal antibody labeled with fluorescent microspheres during the lateral movement, inhibiting its binding with the enrofloxacin-BSA and ciprofloxacin-BSA conjugates on the NC membrane test line. As the content of enrofloxacin and ciprofloxacin in the sample increases, the amount of antibody bound to the test line decreases, and the corresponding test line fluorescence intensity also weakens. Using a dedicated immunofluorescence quantitative analyzer to read the value, the content of enrofloxacin and ciprofloxacin in the sample tissue (fish meat) can be quantitatively determined.

Operating Procedures and Result Interpretation

Bring the unopened test cards and required reagents to room temperature of around 25 degrees Celsius in advance;

Accurately weigh 1.0g of homogenized tissue sample into a 15mL centrifuge tube, add 5mL of extraction solvent (prepared before use: methanol + PBS= 1:1, volume ratio),

vortex for 5 minutes;

Centrifuge at 4000rpm for 5 minutes in a centrifuge;

To ensure the accuracy of the test results, perform a calibration once before each sample measurement. Accurately pipette 100μL of sample dilution liquid and drop it vertically into the sample well of the test card. Allow it to react at room temperature (25℃±2℃) for 10 minutes. Then place the card into the immunofluorescence quantitative analyzer. On the main interface of the instrument, click the “Standard Measurement” button under the “Measurement” menu to perform the calibration;

Take 50μL of the supernatant from step 3) into a 1.5mL centrifuge tube, add 450μL of sample dilution liquid, vortex for 15 seconds. Then accurately pipette 100μL of this mixture and drop it vertically into the sample well of the test card. Allow it to react under the same conditions as in step 4), and start timing;

After 10 minutes of reaction, place the test card into the immunofluorescence quantitative analyzer;

Place the corresponding standard curve smart card in the instrument's induction area. On the main interface, click to read the smart card under the “Measurement” menu, and then click “Sample Measurement” to perform the detection;

After the instrument completes the reading, it will automatically calculate the content of enrofloxacin and ciprofloxacin in the tested sample. You can choose to store and print the results.

Analysis of Each Step

1. Bring the unopened test cards and required reagents to room temperature (24℃±2℃):

Ensure reagent stability and activity: Balancing the reagents to room temperature ensures that their stability and activity are optimized, thereby enhancing the accuracy and reliability of the test.

Minimize the impact of temperature changes on test results: By balancing them to room temperature, the potential influence of temperature differences on the test results can be reduced.

Facilitate operation: At room temperature, the viscosity and fluidity of the reagents are better, making them easier to handle and pipette, reducing errors during the operation.

Prevent crystallization and precipitation: Restoring the reagents to room temperature ensures that they are fully dissolved, preventing crystallization and precipitation, which

in turn guarantees the accuracy of the test.

Improve detection efficiency: At room temperature, the reaction rate of the reagents is usually faster, which helps to improve the efficiency of the test.

2. Accurately weigh 1.0g of homogenized tissue sample into a 15mL centrifuge tube:

Ensure sample homogeneity: This ensures that the sample is uniform, facilitating subsequent extraction and analysis.

3. Add 5mL of extraction solvent (prepared before use: methanol + PBS = 1:1,, volume ratio):

Mix thoroughly: Vortexing for 5 minutes ensures that the sample is well mixed, improving the uniformity of the mixture.

4. Centrifuge at 4000rpm for 5 minutes in a centrifuge:

Separate solids and liquids: This can separate solid particles (such as tissue fragments, cell debris, etc.) from the liquid, making the liquid sample in the supernatant clearer and more suitable for subsequent extraction and analysis.

Concentrate the sample: It allows solid impurities to settle at the bottom, thus concentrating the target analytes (such as enrofloxacin) in the supernatant, enhancing the sensitivity and accuracy of the test.

Remove interfering substances: Interfering substances added during the extraction process, such as anhydrous sodium sulfate, can be removed by centrifugation, reducing their interference with subsequent detection steps.

Improve extraction efficiency: It accelerates the sedimentation of precipitates, shortens the extraction time, and increases the efficiency of the entire detection process.

Prepare for subsequent steps: The supernatant obtained after centrifugation can be directly used for subsequent detection steps.

5. Perform a standard calibration before each sample measurement to ensure the accuracy of the test results:

Add 100μL of sample diluent to the calibration card: This step is crucial for ensuring that the instrument is properly calibrated to provide accurate readings.

6. Take 50μL of the supernatant into a 1.5mL centrifuge tube and add 450μL of sample diluent:

Adjust concentration: The sample diluent adjusts the concentration of enrofloxacin in the sample to a range suitable for detection. This is very important for improving the

sensitivity and accuracy of the test.

SECTION S3 Student Experimental Guidance and Data Recording

Part 1: Micropipette Calibration (via "AquaUncertainty Pal" App)

Student Name(s): _________ Group No.: _______ Date: _____________

Data Recording**:** (Refer to Table S1 Template)

Part 2: Spiked Fish Extract Analysis (Follow "AquaUncertainty Pal" App Guidance)

Volume of extract solution added to test strip (µL): ______ using Pipette ID: P_______

Incubation: Start Time: _____ End Time: _______ Approx. Room Temp (°C): _______

TRFIA Reader Output (Values entered into App):

- ENR Raw Signal / Preliminary Concentration: __________ (Units: [µg/kg])
- CIP Raw Signal / Preliminary Concentration: ___________ (Units: [µg/kg])

Part 3: Results and Analysis (from "AquaUncertainty Pal" App)

Final Quantitative Results:

- ENR Concentration: __________ ± __________ (µg/kg, k=2) (For reference)
- CIP Concentration: __________ ± __________ (µg/kg, k=2) (For reference)
- Total Fluoroquinolones (Total FQs): __________ ± __________ (µg/kg, k=2) (Primary Result)

Analysis of Uncertainty Budget for Total FQs (from App):

- Dominant Source 1: ___________________________ (% Contribution: _____)
- Dominant Source 2: ___________________________ (% Contribution: _____)
- Dominant Source 3 (if prominent): ______________ (% Contribution: _____)

SECTION S4 Assessment Materials and Trainee Survey

The following pre-laboratory and post-laboratory questions are designed to assess student understanding of the core concepts addressed in the "Mobile Application-Assisted Laboratory Exercise for Understanding Measurement Uncertainty in Quantitative Immunoassay." These assessments contribute to the overall evaluation of learning outcomes, as summarized in Table 3 of the main manuscript.

A. Pre-Laboratory Questions (To be completed by students prior to the laboratory session)

1. Distinguish between the metrological concepts of "accuracy" and "precision" as they apply to chemical measurements. (Corresponds to Learning Objective 1)
2. Identify potential sources of systematic and random errors when using a micropipette for volumetric transfers. How do these error types relate to accuracy and precision? (Corresponds to Learning Objective 1)
3. Define "measurement uncertainty." How does it differ conceptually from a measurement "error"? (Corresponds to Learning Objective 1, 5)
4. In a typical multi-step quantitative chemical analysis, enumerate at least three common procedural stages or factors that can contribute significantly to the uncertainty of the final reported result. (Corresponds to Learning Objective 1)
5. Describe the fundamental principle underlying a competitive immunoassay. In such an assay, what is the expected relationship between the concentration of the target analyte in a sample and the magnitude of the measured analytical signal? Provide a brief rationale. (Corresponds to Learning Objective 2, 4)

B. Post-Laboratory Questions (To be completed by students after conducting the experiment and data analysis)

1. Referencing the data from your micropipette calibration (Part 1), including the mean delivered volume and the **app-reported standard uncertainty** $u(V_{\text{pipette}})$ for your P100 (or other specified pipette)
2. Evaluate its performance in terms of accuracy and precision. Justify your evaluation with specific data. (Corresponds to Learning Objective 1)
3. The Time-Resolved Fluorescence Immunoassay (TRFIA) employed in this exercise operates on a competitive binding principle. Provide a concise explanation for the observed inverse relationship between the sample concentration of ENR/CIP and the fluorescence signal intensity measured by

the reader. (Corresponds to Learning Objective 2, 4)

4. State the final quantitative result for Total Fluoroquinolones (Total FQs) obtained from the "AquaUncertainty Pal" application, ensuring it is reported in the correct format: $X_{\text{Total}} \pm U_{\text{Total}}$ (units, k=2). Articulate the metrological meaning of the expanded uncertainty term (U_{Total}) in this context. (Corresponds to Learning Objective 3, 5)
5. Analyze the uncertainty budget chart (similar to Figure 2 in the main manuscript) generated by the "AquaUncertainty Pal" application for your **Total FQs** measurement.
   - Identify the two primary sources that contribute most significantly to the overall expanded uncertainty (U_{Total}) of your result.
   - Propose plausible reasons, based on your experimental execution or understanding of the method, for the prominence of these identified uncertainty sources. (Corresponds to Learning Objective 1, 5)
6. Assume the regulatory limit (MRL) for Total Fluoroquinolones (ENR+CIP) in the relevant aquaculture product is [Specify limit, 100 µg/kg]. Based on your experimentally determined result ($X_{\text{Total}} \pm U_{\text{Total}}$), provide a reasoned conclusion regarding the compliance status of the simulated sample. Clearly state whether it is unequivocally compliant, unequivocally non-compliant, or if the result is inconclusive with respect to the MRL, and justify your conclusion. (Corresponds to Learning Objective 5, 6)
7. Reflecting on the entirety of this laboratory exercise, identify and discuss the specific operational steps or analytical considerations that you now perceive as most critical for ensuring the reliability and minimizing the overall uncertainty of quantitative results obtained from such rapid testing methods. (Corresponds to Learning Objective 1, 6)

C. Answer Key for Instructors(Key points for evaluating student responses. Language should be adapted if used directly by students not in a formal academic program.)

- **Pre-Lab:**
   1. Accuracy: Closeness of agreement between a measured quantity value

and a true quantity value. Precision: Closeness of agreement between indications or measured quantity values obtained by replicate measurements on the same or similar objects under specified conditions (often expressed as SD or RSD).

2. Systematic errors (affect accuracy): incorrect pipette calibration, consistently flawed technique (e.g., incorrect immersion depth). Random errors (affect precision): variations in plunger pressure, minor inconsistencies in handling, environmental fluctuations.

3. Measurement uncertainty: Non-negative parameter characterizing the dispersion of the quantity values being attributed to a measurand, based on the information used. Error: Measured quantity value minus a reference quantity value (true value is idealized and usually unknown). Uncertainty quantifies doubt, not a mistake.

4. Volumetric transfers (pipetting, dilutions), gravimetric measurements (weighing), instrument signal reading variability, calibration standard purity/preparation, sample matrix effects, environmental conditions (temperature).

5. Competitive immunoassay: Analyte in the sample competes with a fixed amount of labeled analyte for a limited number of antibody binding sites. Higher sample analyte concentration results in less labeled analyte binding, leading to a decreased measured signal.

- **Post-Lab:**

1. Accuracy: Compare app-reported mean volume to the set nominal volume. Precision: Evaluate the magnitude of the **app-reported** $u(V_{\text{pipette}})$ (which is directly related to the SD of their replicate weighings); a smaller $u(V_{\text{pipette}})$ indicates higher precision.

2. In a competitive assay, a higher concentration of unlabeled analyte (ENR/CIP from the sample) successfully competes for and occupies more of the limited antibody binding sites on the test strip. This leaves fewer sites available for the labeled detection conjugate (which generates the fluorescence signal) to bind. Consequently, less bound label results in a lower fluorescence signal.

3. The " $\pm U_{\text{Total}}$ " represents the expanded measurement uncertainty, defining an interval [$[X_{\text{Total}} - U_{\text{Total}}, X_{\text{Total}} + U_{\text{Total}}]$] within which the true value of the total fluoroquinolone concentration is believed to lie with a stated level of confidence (typically 95% for k=2). It quantifies the reliability or "believability" of the measured value X_{Total}

4. (Student-specific, based on their app output). Examples: "Uncertainty of Pipette P100 for Dilution" and "Inherent Assay Variability (RSD_assay)". Reasons: "My P200 calibration might have shown a relatively high $u(V_{\text{pipette}})$ due to my technique," or "The kit itself has a documented level of imprecision (RSD_assay) that contributes significantly regardless of my operation."

5. Compare the interval $[X_{\text{Total}} - U_{\text{Total}}, X_{\text{Total}} + U_{\text{Total}}]$ to the MRL. If the entire interval is below the MRL, the sample is compliant. If the entire interval is above the MRL, it is non-compliant. If the MRL falls within the interval, the result is inconclusive; a more precise method would be needed for a definitive judgment.

6. Critical steps include: meticulous micropipette calibration and consistent pipetting technique (especially for small volumes and critical dilutions, as their $u(V_{\text{pipette}})$ directly propagates); adherence to specified incubation times and temperatures; accurate transcription of instrument readings; understanding the limitations (inherent variability) of the rapid test kit itself.

SECTION S5 LC–MS/MS Uncertainty Framework and MRM Parameters

S5.1 Purpose and harmonized reporting

To address reviewer concerns regarding inconsistent uncertainty notation between the QQ method and LC–MS/MS, this section provides a simplified, GUM-consistent framework to estimate the combined standard uncertainty (u_c) for LC–MS/MS results and to report the expanded uncertainty U (k = 2) in a harmonized format. In earlier manuscript versions, the LC–MS/MS result dispersion was shown as "±7.6", which represents the standard deviation (SD, k = 1) across replicate measurements. In the revised submission, LC–MS/MS uncertainty is reported as U (k = 2) to match the QQ reporting convention.

S5.2 Measurement model

For LC–MS/MS quantification using a linear calibration model, the concentration in the final extract (or injected solution) can be expressed as:

$$\hat{C} = \frac{y - b}{a} \quad \text{(S10)}$$

where y is the measured instrument response (typically peak area or area ratio), a is the calibration slope, and b is the intercept. The reported sample concentration in the original matrix (e.g., µg/kg) is obtained by applying the overall sample preparation factor F (including extraction volume, dilution, reconstitution volume, and sample mass):

$$\hat{C}_{sample} = \hat{C} \times F \quad \text{(S11)}$$

In a simplified framework, the combined standard uncertainty of the LC–MS/MS result is assembled from three major contributors: repeatability, calibration, and recovery/matrix effects:

$$u_c = \sqrt{{u_{rep}}^2 + {u_{cal}}^2 + {u_{rec}}^2} \quad \text{(S12)}$$

The expanded uncertainty is then:

$$U = k \times u_c,\ k = 2 \quad \text{(S13)}$$

S5.3 Repeatability component

Repeatability captures variability from independent replicate processing and measurement (e.g., replicate extraction and/or replicate injections). If SD is computed from n independent replicate results of the same material (QC, spiked blank, or sample replicate), then:

$$u_{rep} = SD \quad \text{(S14)}$$

If the reported value is the mean of n replicates and the uncertainty is assigned to the mean,

then:

$$u_{rep,mean} = \frac{SD}{\sqrt{n}} \quad \text{(S15)}$$

S5.4 Calibration component (u_cal)

The calibration uncertainty can be separated into (i) regression/fit uncertainty and (ii) calibrant preparation uncertainty. In a minimal approach, u_fit can be estimated from the regression residuals and expressed as:

$$u_{fit} = \frac{s_{y/x}}{|a|} \quad \text{(S16)}$$

If the calibrant has a certificate-based uncertainty or preparation uncertainty, it can be expressed as a relative standard uncertainty u_rel,std, contributing:

$$u_{std} = \hat{C} \times u_{rel,std} \quad \text{(S17)}$$

Thus, a practical combined calibration component is:

$$u_{cal} = \sqrt{u_{fit}^2 + u_{std}^2} \quad \text{(S18)}$$

S5.5 Recovery / matrix-effect component (u_rec)

If the LC–MS/MS result is not corrected by recovery, the uncertainty from recovery/matrix effects can be represented using validation recovery data. Let recovery values be R_j (as fractions, not %), with mean R̄ and SD_R. A relative standard uncertainty can be defined as:

$$u_{rel,rec} = \frac{SD\_R}{R} \quad \text{(S19)}$$

and then:

$$u_{rec} = \hat{C}_{sample} \times u_{rel,rec} \quad \text{(S20)}$$

S5.6 Reporting format used in this study

In the revised manuscript, LC–MS/MS results are reported with expanded uncertainty as:

$$\hat{C}_{sample} \pm U(k = 2) \quad \text{(S22)}$$

List of Figure

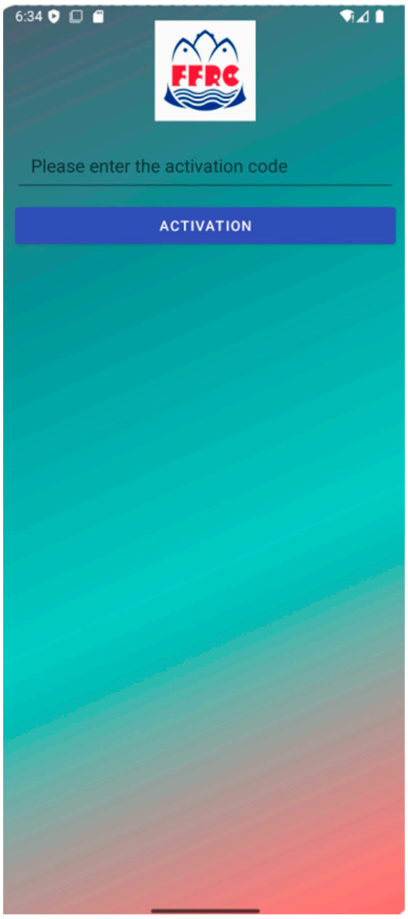

Figure S1. Activation Function and App Login Screen

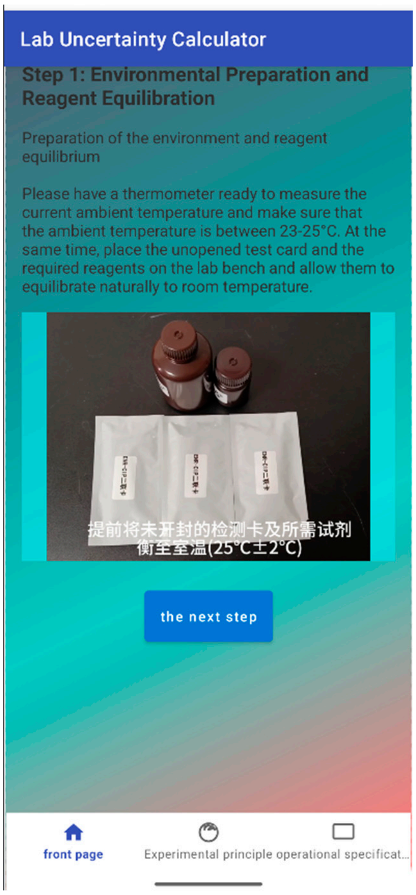

Figure S2. Environmental Equilibrium Page

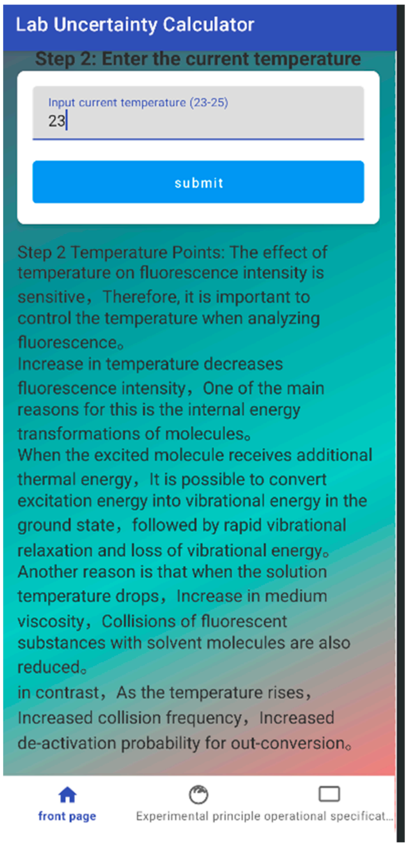

Figure S3. Temperature Input and Environmental Control Prompt

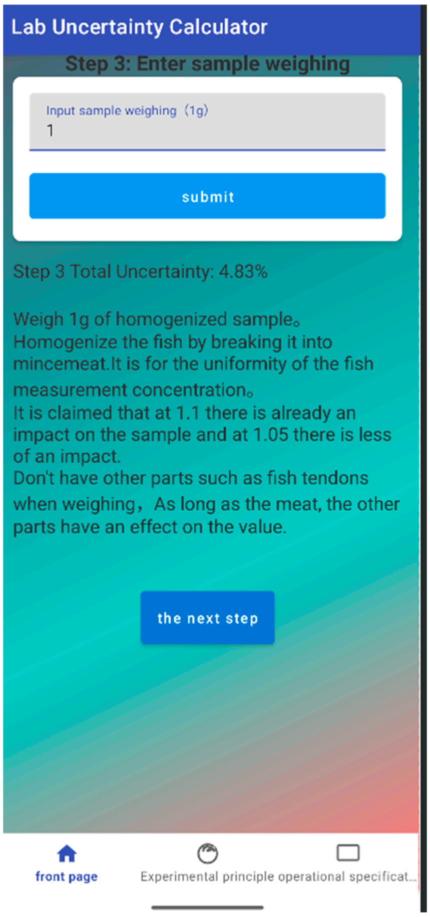

Figure S4. Sample Weighing Data Entry Interface

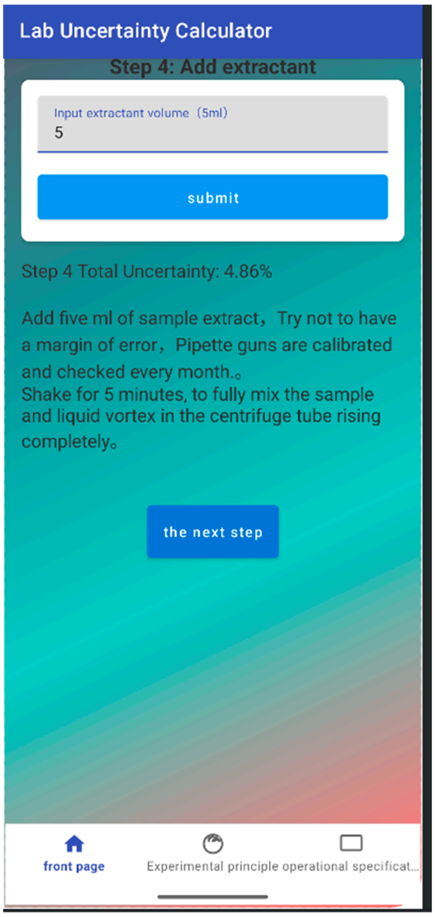

Figure S5. Reagent Addition Volume Entry Interface

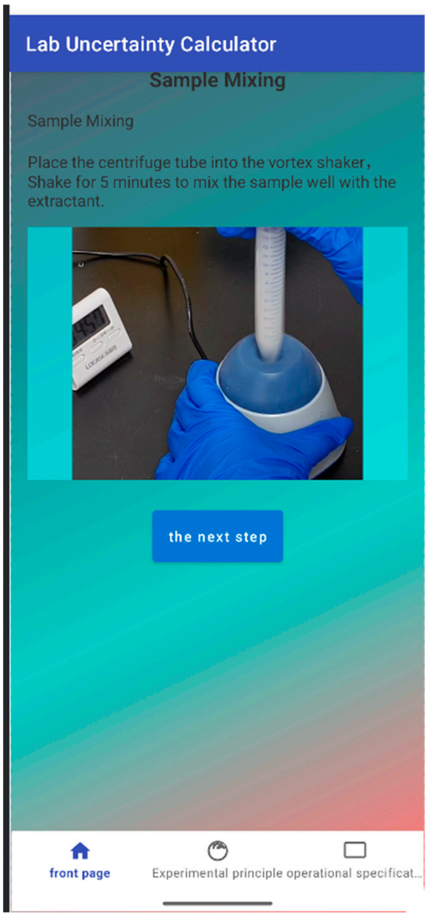

Figure S6. Sample Vibration

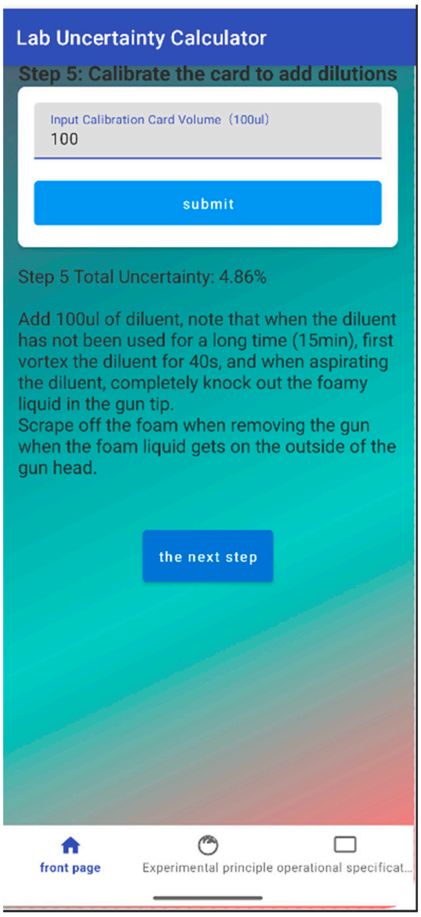

Figure S7. Calibration Curve

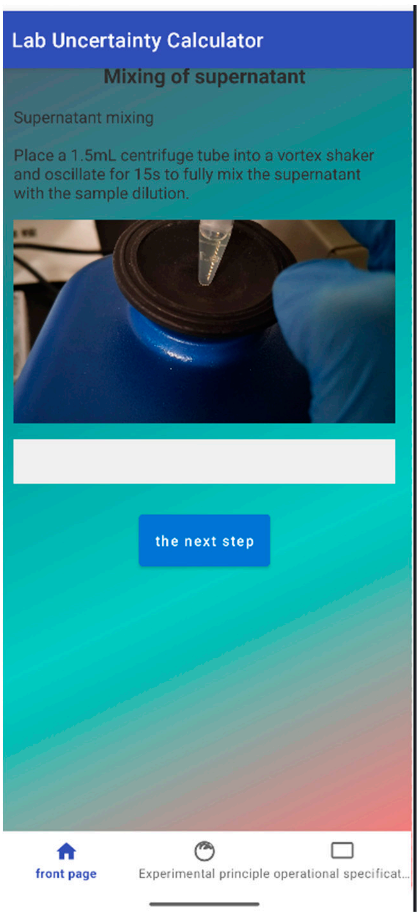

Figure S8. Mixing the Test Solution

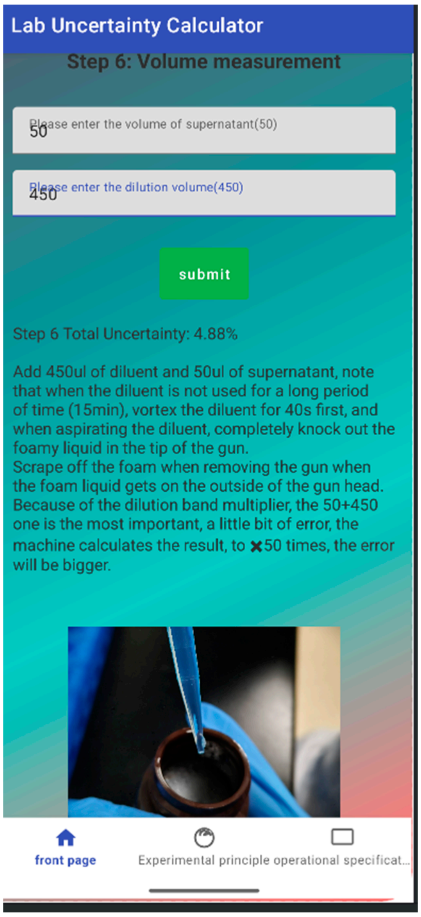

Figure S9. Dilution and Spotting Instructions

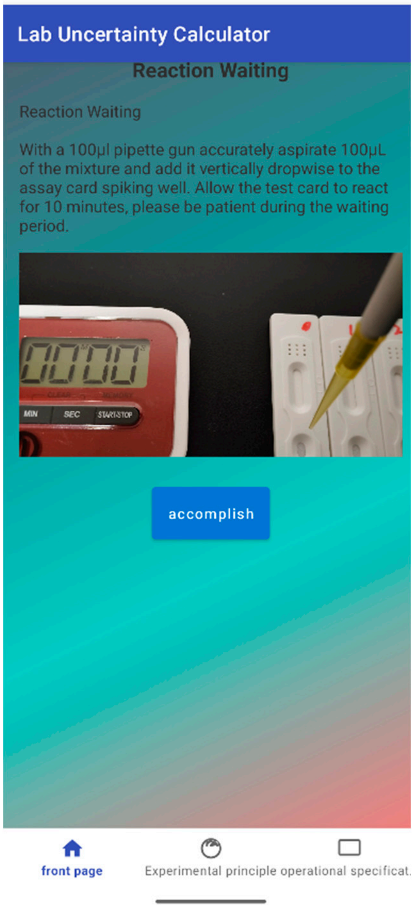

Figure S10. Reagent Card Reaction

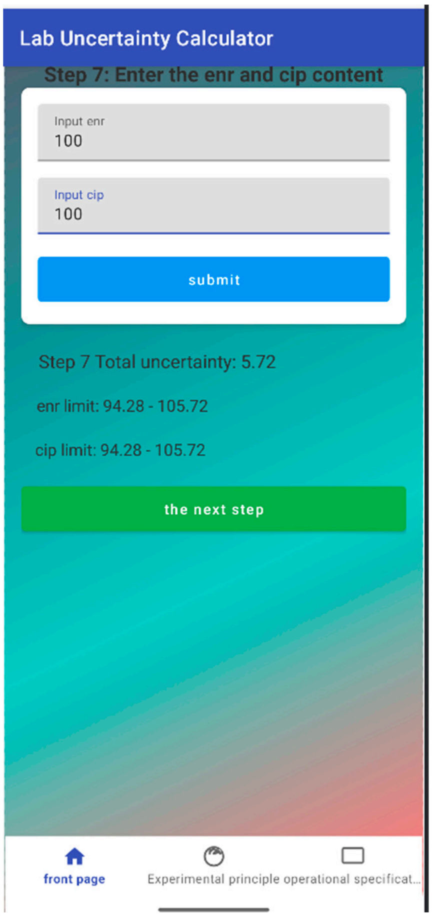

Figure S11. Concentration Result Page

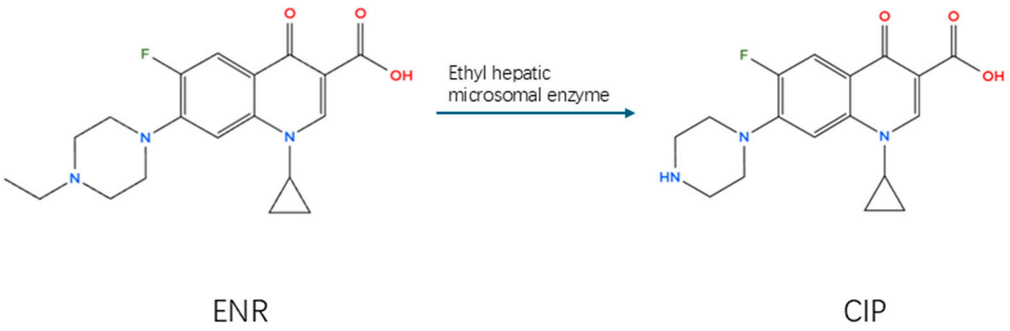

Figure S12. Chemical structures of enrofloxacin (ENR) and ciprofloxacin (CIP).

List of Tables

Table S1. Performance comparison between the Quick Quantitative (QQ) method and the standard LC-MS/MS method. This table provides the summarized data supporting the analysis in Section 3.2 and the visualization in Figure 4 of the main manuscript. Values are X ± U (k = 2) unless otherwise stated. For LC–MS/MS, the notation ± 7.6 refers to the standard deviation across replicates

Parameter	LC-MS/MS	QQ method + AquaUncertainty Pal
Mean recovery (μg/kg)	199.7 ± 7.6	188.1 ± 23.5
Expanded uncertainty (U, k=2)	15.3 (7.7%)	23.5 (12.5%)
Time per sample (min)	120	25
Operator skill requirement	High (professional background needed)	Low (Application-guided)

Table S2. Comparison of pipetting precision (RSD %) for 20 technicians before and after training. This table contains the detailed data for each participant, supporting the analysis in Section 3.3 and the visualizations in Figure 5 of the main manuscript.

Participant ID	100μL Pre-training RSD(%)	100μL Post-training RSD(%)	1000μL Pre-training RSD(%)	1000μL Post-training RSD(%)
1	3.2	1.8	2.1	1.2
2	4.5	0.9	2.8	0.7
3	2.9	1.5	1.9	0.5
4	4.1	2.3	2.5	1.3
5	3.7	1.9	2.3	1.1
6	5.1	2	3.3	1.4
7	4.3	1.7	2.7	0.9
8	3.9	1.6	2.2	1
9	4.7	2.1	3.1	1.5
10	3.5	1.4	2	0.8
11	4.9	2.2	2.9	1.3
12	3.3	1.5	2.4	0.9
13	4	1.8	2.6	1
14	5	2.4	3.2	1.6
15	3.8	1.7	2.1	1.1
16	4.6	2	2.7	1.2
17	3.6	1.3	1.8	0.7
18	4.4	1.9	2.5	1
19	5.2	2.5	3.4	1.7
20	3.4	1.2	1.9	0.6

Table S3. Raw data from multiple independent comparative experiments between LC–MS/MS and the QQ method.

Sample ID	LC-MS			Card			
	ENR	CIP	ENR+ CIP	ENR	CIP	ENR+ CIP	Recovery Rate (%)
24.7.10sample 1	105	90.7	196	124.1	97.7	221.8	113.2%
24.7.10sample 2	121	108	229	114.3	73.8	188.1	82.1%
24.7.10sample 3	106	88.6	194.6	117.9	69.8	187.7	96.5%
24.7.10sample 4	112.6	100	212.6	134.3	112.8	247.1	116.2%
24.7.10sample 5	105	90.5	195.5	170.7	167.4	338.2	173.0%
24.7.10sample 6	305	291	596	239.2	272.0	511.3	85.8%
24.7.10sample 7	89.5	65	154.5	106.5	64.8	171.3	110.9%
24.7.10sample 8	92	74	166	119.6	79.4	199.0	119.9%
24.7.10sample 1	105	90.7	196	122.5	81.8	204.2	104.2%
24.7.10sample 6	305	291	596	239.0	301.4	540.4	90.7%
24.7.10sample 7	89.5	65	154.5	91.5	63.5	155.1	100.4%
24.7.10sample 1	105	90.7	196	120.8	79.9	200.7	102.4%
24.7.10sample 6	305	291	596	211.6	307.7	519.3	87.1%
24.7.10sample 7	89.5	65	154.5	86.0	49.0	135.0	87.4%
24.7.10sample 1	105	90.7	196	116.6	88.4	205.1	104.6%
24.7.10sample 4	112.6	100	212.6	127.8	62.9	190.8	89.7%
24.7.10sample 8	92	74	166	105.5	62.5	167.9	101.2%
24.7.10sample 2	121	108	229	137.6	92.5	230.1	100.5%
24.7.10sample4	112.6	100	212.6	132.6	101.0	233.6	109.9%
24.7.10sample 5	105	90.5	195.5	150.7	128.4	279.1	142.8%
24.7.10sample 8	92	74	166	126.7	91.3	218.0	131.3%
24.7.10sample4	112.6	100	212.6	117.3	103.7	221.0	104.0%
24.7.10sample 5	105	90.5	195.5	123.3	93.2	216.5	110.7%
24.7.10sample 8	92	74	166	88.7	48.9	137.5	82.8%
24.10.18sample1	1	27.1	78.1	74.5	17.6	92.1	117.9%
24.10.18sample2	6	23.6	99.6	92.5	27.0	119.4	119.9%
24.10.18sample 3	169.4	21.4	190.8	131.4	36.7	168.1	88.1%
24.10.18sample4	382.3	27	409.3	208.8	92.3	301.1	73.6%
24.7.10sample5	105	90.5	195.5	152.0	116.1	268.2	137.2%
24.7.10sample8	92	74	166	114.3	73.4	187.7	113.0%

Table S4. Source-resolved uncertainty budget for QQ immunoassay

**Source (i)**	**Symbol**	**Dist.**	**Sensitivity c_i**	**u_i (μg/kg)**	**u_i (% of 188.1)**	**Contribution to u_{c2} (%)**
Lab Card Uncertainty	u_L	normal	1	8.12	4.31%	47.71
Extraction / recovery	u_R	normal	1	6.89	3.66%	34.4
Weighing	u_M	normal	1	3.93	2.09%	11.2
zero-card Calibration	u_Z	normal	1	2.9	1.54%	6.09
Calibration / curve fit	u_F	normal	1	0.91	0.48%	0.6

Table S5. Programmed MRM channels and MS parameters for the quinolone LC–MS/MS acquisition function (ESI+), transcribed from the instrument method screen (method label: PAX).

Function label (as displayed)	Channel #	Parent (m/z) (Q1)	Daughter (m/z) (Q3)	Dwell (s)	Dwell (ms)	Cone (V)	Collision (V)
PAX	1	435.3000	167.1000	0.025	25	34	14
PAX	2	435.3000	344.3000	0.025	25	40	20
PAX	3	435.3000	362.0000	0.025	25	40	20
PAX	4	435.3000	402.4000	0.025	25	40	20
PAX	5	435.3000	420.1000	0.025	25	34	24

Note: The method screen shows "IntelliStart Generated" for selected channels, indicating software-assisted optimization of parameters. The label "PAX" refers to the function name displayed in the current instrument method for the quinolone MRM acquisition.

Observed transition during processing (supporting evidence):

Analyte	Observed MRM transition (m/z)	Ionization	Evidence source
ENR	360.1 → 72.1	ESI+	TargetLynx channel display (screenshot)
